# Under-utilization of Narrow-Spectrum Antibiotics in the Ambulatory Management of Pediatric UTI: A Single-Center Experience

**DOI:** 10.3389/fped.2021.675759

**Published:** 2021-08-16

**Authors:** Philip Lee, Mimi Kim, Betsy C. Herold, Vijaya L. Soma

**Affiliations:** ^1^Division of Pediatric Infectious Disease, The Children's Hospital at Montefiore, Bronx, NY, United States; ^2^Department of Pharmacy, The Children's Hospital at Montefiore, Bronx, NY, United States; ^3^Division of Biostatistics, Department of Epidemiology & Population Health, Albert Einstein College of Medicine, New York City, NY, United States

**Keywords:** urinary tract infection, pediatric, antibiotic, outpatient, school health

## Abstract

**Objective:** There are urinary tract infection (UTI) guidelines for treatment of patients <2 years old, but there is a paucity of data for other pediatric age groups including the potential role for stewardship to reduce prescription of broad-spectrum antibiotics. We assessed practice patterns for the diagnosis and empiric treatment of UTI for outpatient and school health sites affiliated with a large urban pediatric medical center. We hypothesized that outpatient providers under-utilize narrow-spectrum antibiotics, such as first-generation cephalosporins, for uncomplicated UTI.

**Study Design:** Retrospective study from December 1st, 2015 to May 31st, 2016.

**Results:** The study population included 903 children (70.1% female) with a median age of 11 years, evaluated in an outpatient clinic (*n* = 780, 86.4%) or school health site (*n* = 123, 13.6%). *E. coli* was the most common urinary pathogen (50.9%) and 92.6% of *E. coli* isolates were susceptible to cephalexin. However, cephalexin was prescribed empirically for only 12.8% of patients. In contrast, sulfamethoxazole-trimethoprim was commonly prescribed, but only 79% of *E. coli* isolates were susceptible. Antibiotics were discontinued in only three of 48 children who had negative urine cultures.

**Conclusions:** Cephalexin may be the most appropriate first-line choice for management of outpatient UTI for our patient population. Antibiotics were rarely discontinued for those with negative urine cultures. Antibiotic stewardship in the outpatient setting could reduce unnecessary antibiotic exposure in the management of pediatric UTI.

## Introduction

Urinary tract infection (UTI) is a common outpatient pediatric diagnosis. There are guidelines for the diagnosis and management of uncomplicated UTI in children 2–24 months of age but not for older children and adolescents (1, 2). The gold standard for diagnosis is bacterial culture with antibiotic selection based on susceptibility testing. However, results of these diagnostic tests are not immediately available and thus clinicians face the dilemma of whether to empirically treat for a presumptive UTI or delay treatment in a symptomatic patient. Some advocate using urinary nitrites and/or leukocyte esterase on a dipstick to guide decisions about whether to start empiric therapy, but the sensitivity and specificity of these rapid point-of-care tests are variable (2–5). Similarly, quantification of white blood cells and bacteria by microscopy is rarely available in outpatient settings and the sensitivity and specificity of microscopy ranges from 50–96% (2–5).

If a clinician elects to start antibiotic treatment prior to having culture and susceptibility results, empiric therapy is usually based on community patterns of bacterial resistance (1, 6, 7). Mathematical modeling studies suggest that drugs such as amoxicillin, sulfamethoxazole-trimethoprim (SXT) and ciprofloxacin should not be prescribed for empiric therapy if local resistance rates for common urinary pathogens such as *Escherichia (E.) coli* exceed 20% (8, 9). *E. coli* rates of resistance in developed nations have been reported as high as 50 and 30% for SXT and amoxicillin, respectively (10). Studies in adults have shown that treatment of community-acquired *E. coli* UTIs with either SXT or ciprofloxacin is associated with recurrent UTIs and development of resistance within 6–12 months of initial presentation (11, 12). However, similar data in pediatrics is limited.

Given the paucity of data in children, particularly those over 2 years of age, we conducted a retrospective study to assess the practice patterns for diagnosis and empiric treatment of pediatric UTI in a large urban outpatient network consisting of traditional ambulatory sites and school-based health centers. Urine culture results and susceptibility patterns were reviewed. We hypothesized that outpatient providers under-utilize narrow-spectrum antibiotics, defined here as penicillins and first-generation cephalosporins, compared to other, broader-spectrum antibiotics for uncomplicated UTI (13, 14).

## Methods

### Study Design

A retrospective study of patients >2 years and under 21 years of age who sought care in one of 54 outpatient sites associated with The Children's Hospital at Montefiore, Bronx, NY between December 2016 and June 2017 and had an ICD-9 code associated with UTI at the visit (ICD-9 codes 599, 791.9, 788.1, 788.41, 788.63) was conducted. Sites included 17 school health programs and 37 pediatric clinics. Demographics, clinical symptoms, urine testing results, prescribed antibiotics, and related return visits within 6 months of the initial encounter were extracted from the electronic medical record (EMR). Patients were excluded if they were treated with an antimicrobial for a condition other than UTI, were pregnant, had congenital genitourinary abnormalities, or had a past positive urine culture since birth recorded in the EMR. Institutional Review Board approval for the study was obtained from the Albert Einstein College of Medicine Office of Human Research Affairs.

### Definitions

A positive urine screen was defined as a dipstick with detectable leukocyte esterase or nitrites or urine with microscopic findings of ≥10 white blood cells (WBC) per high-powered-field (HPF). All culture and susceptibility testing were performed by the Montefiore Clinical Microbiology Lab. A positive urine culture was defined as having ≥100,000 colony- forming units (CFU)/mL of a single bacterium from a cleanly voided specimen. If two or more organisms were present, the culture was included only if one was present at ≥100,000 CFU/mL and the other was <10,000 CFU/mL. Testing was performed using the Phoenix 100 instrument with automated dilution and software version 6.35A (Becton, Dickinson and Company, Franklin Lakes, NJ) by the Montefiore Medical Center Microbiology Department. Return visits to a clinic were categorized as those occurring within 6 months from the initial visit for a new UTI symptom.

### Statistical Analyses

Positive predictive value (PPV) was defined as proportion of true cases (based on positive urine culture) among all participants with a positive screening test and negative predictive value (NPV) was defined as true non-cases (negative urine culture) among all participants with a negative screening test. Chi square of independence and Fisher's exact tests were used to compare groups with respect to binary outcomes. Continuous variables were compared using Student *t*-test. The Kaplan-Meier method and log rank test were used to estimate and compare between antibiotic groups the distribution of time to clinic return for recurrent UTI symptoms. Descriptive statistics were used when comparing demographics. A two-sided *p-*value of <0.05 was considered statistically significant. All analyses were performed using GraphPad and SAS.

## Results

There were 1,199 patients with an ICD-9 code associated with UTI over the study period and 1,138 had a retrievable EMR. The following patients were excluded: 33 that were treated with an antimicrobial for other conditions (including 22 diagnosed with sexually transmitted infection or fungal vulvovaginitis), 21 who were ≤2 years old, and 181 patients with a previous positive urine culture, yielding a final study population of 903 patients. The majority (*n* = 780, 86.4%) were seen at an outpatient clinic and 123 (13.6%) were evaluated at a school health clinic. The median age of the overall study cohort was 11 years (SD: ±5.9, range 2–21 years). The patients from school health clinics were significantly older than those from outpatient clinics, 16 ± 3 (median ± SD) vs. 9 ± 6 years, respectively (*p* < 0.001). Other demographic characteristic detailed in Table 1 reflect the community population with respect to race and ethnicity.

**Table 1:** Subject demographics.

	**# Patients (*n* = 903)**
Sex, *n* (%)
Male	270 (29.9%)
Female	633 (70.1%)
Race, *n* (%)
American Indian or Alaska Native	7 (0.8%)
Asian	15 (1.7%)
Black	259 (28.7%)
White	67 (7.4%)
Hispanic	350 (38.8%)
Other	77 (8.5%)
Declined to Answer	128 (14.2%)
Median age in years, (±SD)	11 ± 5.9
Outpatient Sites
Clinical, *n* (%)	780 (86.4%)
School Health, *n* (%)	123 (13.6%)

**Figure 1:**
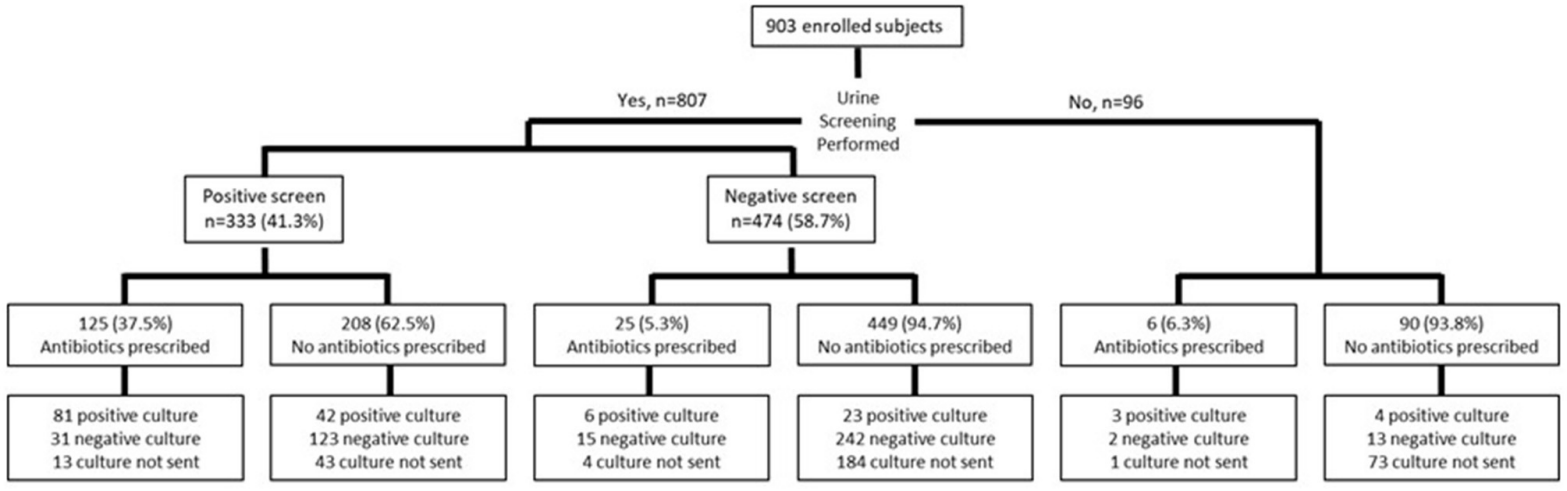
Testing and empiric treatment in pediatric outpatients diagnosed with UTI.

A urine screen was performed in 89.4% (*n* = 807) of patients; 473 had a point of care dipstick, 129 had urine microscopy ordered and performed in the microbiology laboratory and 205 had both screening tests (Figure 1). Urine cultures were obtained in a majority of patients who had a screening test performed (563/807, 69.8%), but were more likely to be sent in those with a positive compared to a negative screen (277/333, 83.1% vs. 286/474, 60.3%, *p* < 0.001). A culture was also sent in 22/96 (22.9%) of those who had no screening test performed. The urine culture was positive in 123/277 (36.9%) with a positive urine screen (PPV: 44.4%, 95% CI: 40.8%, 48.1%), and 30/286 (6.3%) with a negative screen (NPV: 89.5%, 95% CI: 86%, 92.2%). Among those who had no screening test performed, 7/96 (4.3%) had a positive urine culture.

**Figure 2:**
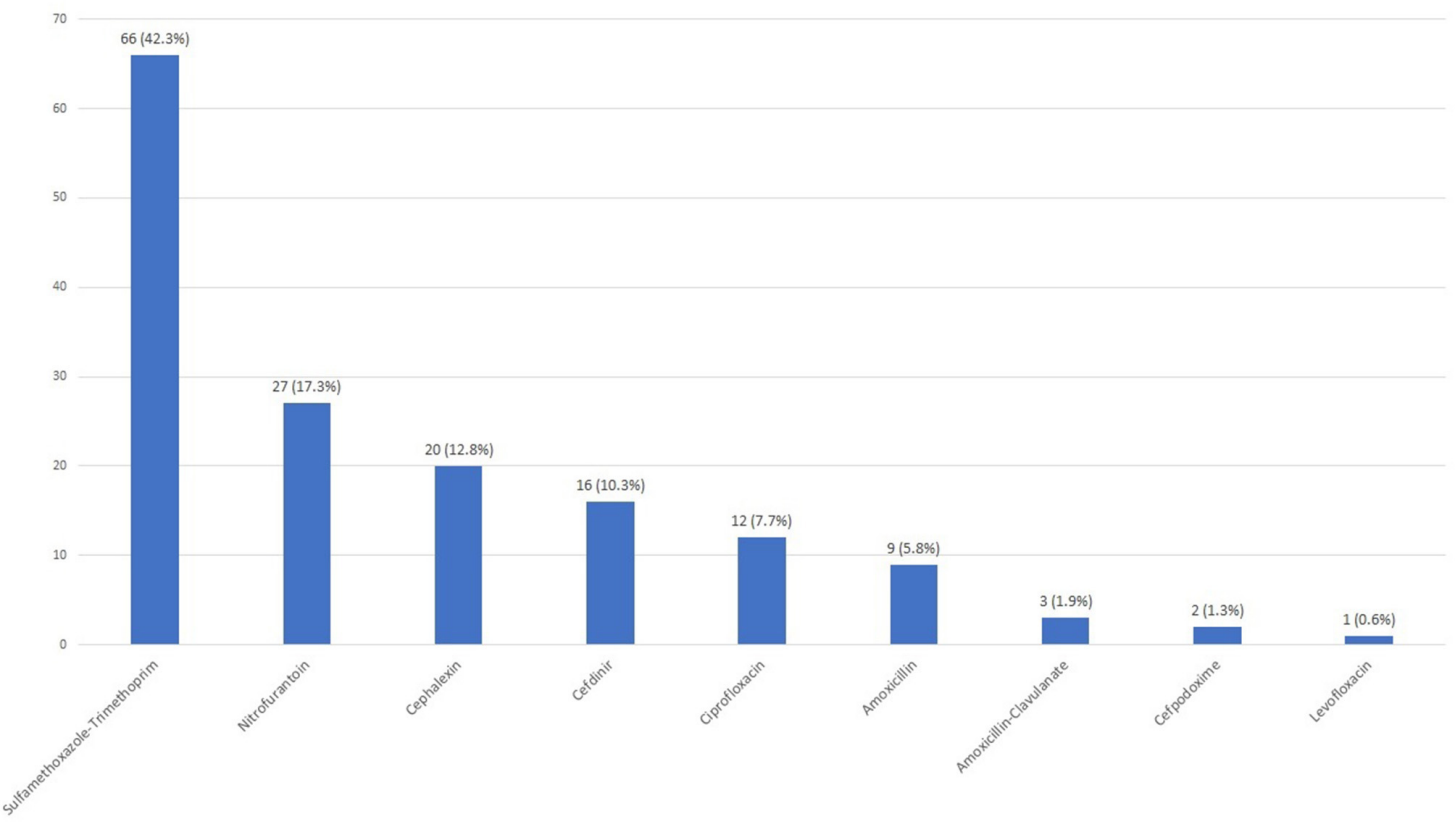
Empiric antibiotic selection. Each bar shows number of patients prescribed in the indicated antibiotic with *n* (%) noted above.

Empiric antibiotics were prescribed more frequently in those with a positive screen (124/333, 37.2%) compared to those with a negative screen (25/474, 2.3%) (*p* < 0.001) (Figure 1). The most commonly prescribed empiric antibiotics were SXT (42.3%), nitrofurantoin (17.3%), cephalexin (12.8%) and ciprofloxacin (7.7%) (Figure 2). School health providers prescribed SXT at a higher rate (65.5%) than other outpatient clinicians (37%), *p* < 0.01 (Table 2).

**Table 2:** Empiric antibiotic choice, by outpatient site, *n* (%).

**Antibiotic**	**School health (*N* = 29)**	**Outpatient (*N* = 127)**	***p*-value**
Cephalexin	1 (3.4%)	19 (15%)	0.1262
Sulfamethoxazole-trimethoprim	19 (65.5%)	47 (37%)	0.0066
Nitrofurantoin	6 (20.7%)	21 (16.5%)	0.5918
Ciprofloxacin	2 (6.9%)	10 (7.9%)	1
Amoxicillin	1 (3.4%)	8 (6.3%)	1
Other	0	22 (17.3%)	–

**Figure 3:**
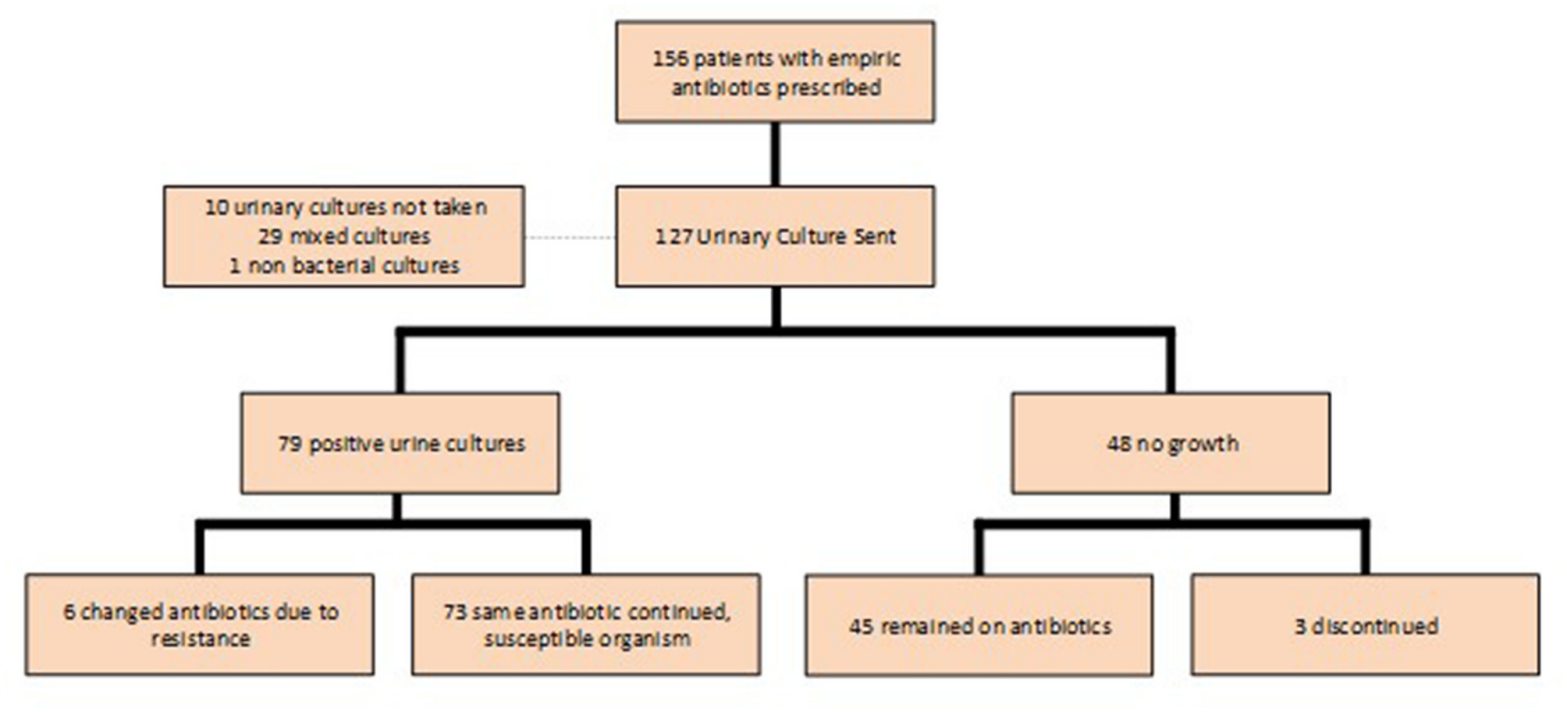
Impact of urine culture result on prescribed treatment.

**Figure 4:**
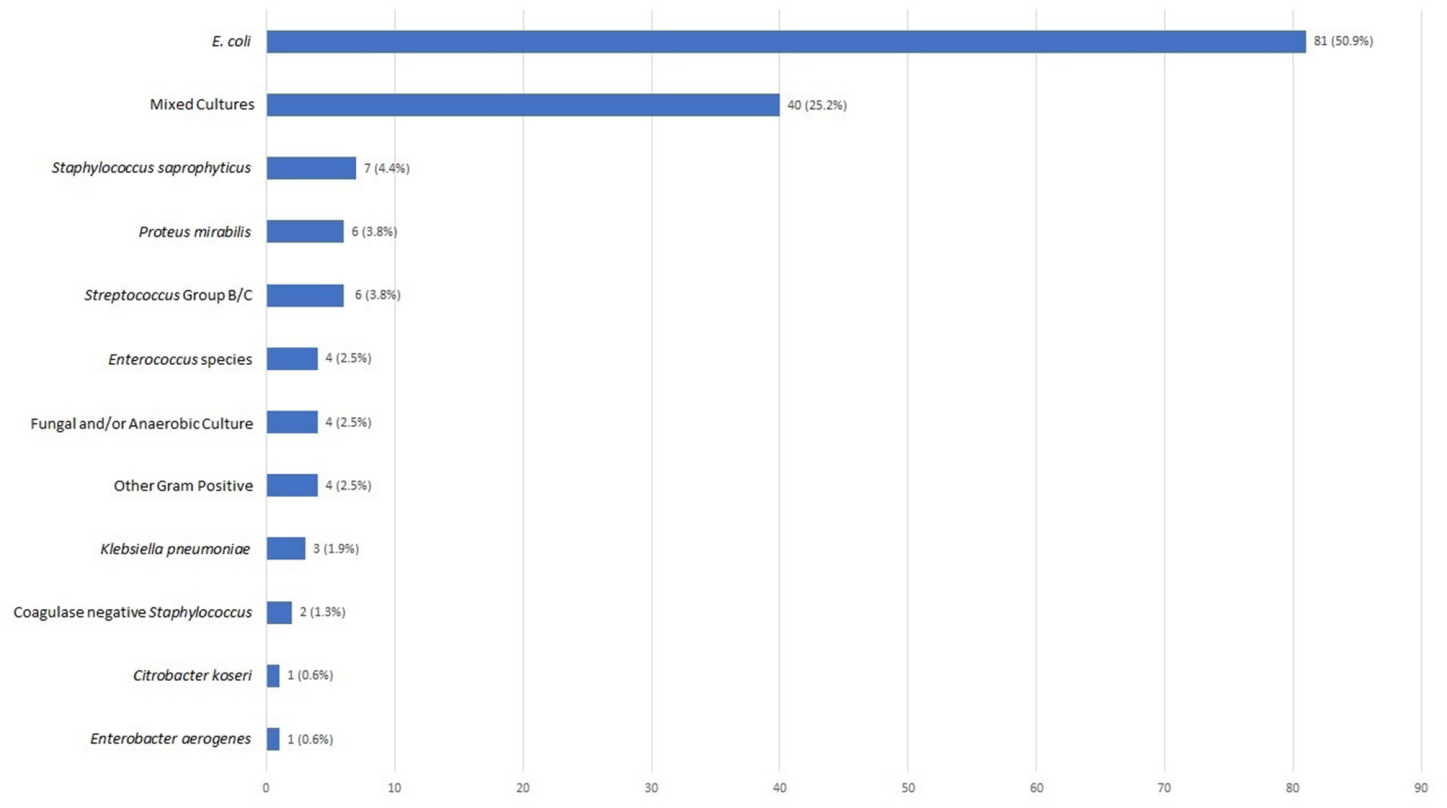
Urinary pathogens identified (*N* = 159). Bar chart reflecting cultured urine pathogens as a percentage of all positive cultures.

Urine cultures were sent in 127 of the 159 (79.9%) patients who were prescribed empiric antibiotics; 79 (62.2%) of the cultures were positive (Figure 3). *E. coli* was the most common pathogen identified and accounted for 50.9% of positive cultures (Figure 4). Other isolates identified included mixed cultures (25.2%), various gram-positive bacteria (14.5%), *Proteus mirabilis* (3.6%), and *Klebsiella pneumoniae* (1.9%).

Based on 2016 Clinical and Laboratory Standards Institute (CLSI) breakpoints (13), rates of *E. coli* susceptibility were highest for cephalexin (92.6%), nitrofurantoin (98.8%) and ciprofloxacin (93.8%) and lowest for SXT (79.8%), tetracycline (76.2%), ampicillin-sulbactam (64.3%) and ampicillin (61.9%). Only 2.5% of *E. coli* isolates produced extended-spectrum beta lactamases (ESBL) based on ceftriaxone susceptibility (13) (Table 3).

**Table 3:** *E. coli* susceptibility to antibiotics across all age groups.

	**Number of susceptible isolates (Total *N* = 81)**
Cefazolin^†^	75 (92.6%)
SXT	64 (79%)
Ampicillin	51 (63%)
Ampicillin-Sulbactam	52 (64.2%)
Ceftriaxone	79 (97.5%)
Ciprofloxacin	76 (93.8%)
Gentamicin	72 (88.9%)
Nitrofurantoin	80 (98.8%)
Tetracycline	61 (75.3%)

† *Susceptibility refers to the new cefazolin breakpoint to treat E. coli, K. pneumoniae and P. mirabilis UTIs*.

There were seven cases in which the bacteria isolated was not susceptible to the empiric antibiotic prescribed and the antibiotic was appropriately changed in all but one case. Complete discontinuation of the empiric antibiotic in the setting of a negative urine culture, however, was infrequent. There were 48 patients in whom urine cultures yielded no growth, but 45 (93.8%) continued therapy; only three had treatment discontinued. Two patients who were continued on therapy were prescribed a different antibiotic because of persistent symptoms (Figure 3).

**Figure 5:**
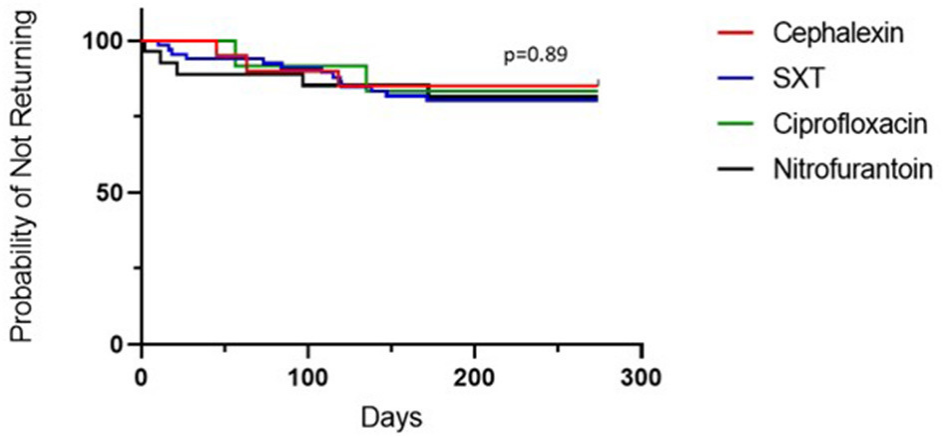
Percentage of patients who did not return to the clinic for recurrent and/or persistent UTI symptoms in the 6 months following diagnosis, by antibiotic. Kaplan-Meier estimates for patients receiving each antibiotic.

Treatment with cephalexin was associated with the lowest rate of clinic return for recurrent or persistent symptoms within 6 months of initial presentation, but this did not differ significantly compared to other antibiotics as shown in the Kaplan-Meier plot (Figure 5). Return rates were 15% (3/20) for cephalexin, 19.7% (13/66) for SXT, 18.5% (5/27) for nitrofurantoin and 16.7% (2/12) for ciprofloxacin. There were no returns within the 1st month when patients were treated with cephalexin or ciprofloxacin.

## Discussion

We hypothesized that outpatient pediatric providers under-utilize narrow-spectrum antibiotics for uncomplicated UTI and found this to be true of outpatient practices affiliated with our institution. A high percentage of the *E. coli* isolates were susceptible to cephalexin, but this antibiotic was infrequently prescribed as the empiric choice in both the school health and outpatient clinic setting. Cephalexin was the least prescribed in the school health setting and the third least in the outpatient setting. Physicians prescribed SXT or nitrofurantoin most often. Moreover, even after organism identification and susceptibility results were available, treatment was only changed if the organism was resistant to the initial antibiotic; narrowing of antibiotic treatment did not occur. The latter is understandable since the treatment course is relatively short and changing a prescription would result in increased patient costs. Cephalexin was also associated with a low rate of symptom recurrence in this study compared to SXT or nitrofurantoin. Cephalexin may also improve adherence as it has a more favorable drug frequency (twice per day vs. four times per day with nitrofurantoin) (5, 14, 15). Taken together, the results suggest that a first-generation cephalosporin might prove to be a better empiric first-line therapy for pediatric outpatient UTI compared to SXT, nitrofurantoin, or ciprofloxacin.

The finding that providers preferentially selected SXT as the empiric antibiotic for UTI is similar to the adult literature, where SXT was empirically prescribed even when there were higher rates of resistance prevalent in the community (16, 17). The Infectious Diseases Society of America (IDSA) guidelines recommend that an antibiotic not be empirically prescribed if local resistance rates exceed 20% (6). The overall *E. coli* resistance rate to SXT was 20.2% in the current study. These findings highlight the need for outpatient antimicrobial stewardship interventions and education.

Antibiotics were discontinued in only three of the 48 (6.3%) patients with negative urine cultures, which is consistent with an earlier study in a pediatric urgent care network which found that antibiotics were discontinued in only 4% of patients with negative urine cultures. The authors introduced a quality improvement educational intervention, which led to a greatly increased discontinuation rate of 84% (18). It is unclear what barriers may prevent providers from discontinuing antibiotics in the context of negative urine cultures and this area deserves more study, in order to design appropriate educational interventions and support mechanisms. Our findings suggest that quality improvement interventions designed both at discontinuing antibiotics when cultures are negative but also changing the choice of empiric front-line therapy (when appropriate) are needed.

It is important to point out that while *E. coli* was the most commonly identified pathogen in our study, as expected, the frequency (50%) was lower than the 78 and 80% previously reported (3, 19, 20). Our second most common microbiological result was mixed cultures, which could be attributed to suboptimal techniques used to collect urinary samples in the outpatient setting.

Advantages to the current study include the large patient population including school-based clinics and the use of a single microbiology laboratory. Limitations include the retrospective nature, reliance on medical record documentation to identify patients, limited information regarding duration of antibiotic treatment, provider variability in use of rapid screening tests and potential variability in urine collection methods. It is possible that we did not capture all recurrences as patients may have sought care outside this healthcare system. In addition, the findings may not be generalizable to other communities where susceptibility patterns may differ. It is, however, very reassuring that in a large urban community, the majority of isolates were *E. coli* that were susceptible to cephalexin. Our findings suggest that there is a need for antibiotic stewardship interventions in the outpatient setting to promote the empiric use of narrow-spectrum antibiotics for pediatric patients with UTI symptom.

## Data Availability Statement

The raw data supporting the conclusions of this article will be made available by the authors, without undue reservation.

## Author Contributions

BH, VS, and PL were involved in the development of the concept and the design of the review, acquisition and gathering of data, statistical analysis, interpretation of results, and drafting and revising the manuscript. MK contributed to study design, reviewed the statistical analysis and performed additional analyses, and reviewed and edited the final manuscript. All authors approve the final manuscript as submitted.

## Conflict of Interest

The authors declare that the research was conducted in the absence of any commercial or financial relationships that could be construed as a potential conflict of interest.

## Publisher's Note

All claims expressed in this article are solely those of the authors and do not necessarily represent those of their affiliated organizations, or those of the publisher, the editors and the reviewers. Any product that may be evaluated in this article, or claim that may be made by its manufacturer, is not guaranteed or endorsed by the publisher.

## Abbreviations

SXT: Sulfamethoxazole-trimethoprim
UTI: urinary tract infection
UA: urinalysis.
